# Reduced thoracolumbar fascia shear strain in human chronic low back pain

**DOI:** 10.1186/1471-2474-12-203

**Published:** 2011-09-19

**Authors:** Helene M Langevin, James R Fox, Cathryn Koptiuch, Gary J Badger, Ann C Greenan- Naumann, Nicole A Bouffard, Elisa E Konofagou, Wei-Ning Lee, John J Triano, Sharon M Henry

**Affiliations:** 1 Department of Neurology, University of Vermont, Burlington VT, USA; 2 Department of Orthopedics & Rehabilitation, University of Vermont, Burlington VT, USA; 3 Department of Medical Biostatistics, University of Vermont, Burlington VT, USA; 4 Orthopaedic Specialty Center, Fletcher Allen Health Care, Burlington VT, USA; 5 Departments of Biomedical Engineering and Radiology, Columbia University, New York, NY, USA; 6 Canadian Memorial Chiropractic College, Toronto, ON, Canada; 7 Department of Rehabilitation & Movement, Science, University of Vermont, Burlington VT, USA

## Abstract

**Background:**

The role played by the thoracolumbar fascia in chronic low back pain (LBP) is poorly understood. The thoracolumbar fascia is composed of dense connective tissue layers separated by layers of loose connective tissue that normally allow the dense layers to glide past one another during trunk motion. The goal of this study was to quantify shear plane motion within the thoracolumbar fascia using ultrasound elasticity imaging in human subjects with and without chronic low back pain (LBP).

**Methods:**

We tested 121 human subjects, 50 without LBP and 71 with LBP of greater than 12 months duration. In each subject, an ultrasound cine-recording was acquired on the right and left sides of the back during passive trunk flexion using a motorized articulated table with the hinge point of the table at L4-5 and the ultrasound probe located longitudinally 2 cm lateral to the midline at the level of the L2-3 interspace. Tissue displacement within the thoracolumbar fascia was calculated using cross correlation techniques and shear strain was derived from this displacement data. Additional measures included standard range of motion and physical performance evaluations as well as ultrasound measurement of perimuscular connective tissue thickness and echogenicity.

**Results:**

Thoracolumbar fascia shear strain was reduced in the LBP group compared with the No-LBP group (56.4% ± 3.1% vs. 70.2% ± 3.6% respectively, p < .01). There was no evidence that this difference was sex-specific (group by sex interaction p = .09), although overall, males had significantly lower shear strain than females (p = .02). Significant correlations were found in male subjects between thoracolumbar fascia shear strain and the following variables: perimuscular connective tissue thickness (r = -0.45, p <.001), echogenicity (r = -0.28, p < .05), trunk flexion range of motion (r = 0.36, p < .01), trunk extension range of motion (r = 0.41, p < .01), repeated forward bend task duration (r = -0.54, p < .0001) and repeated sit-to-stand task duration (r = -0.45, p < .001).

**Conclusion:**

Thoracolumbar fascia shear strain was ~20% lower in human subjects with chronic low back pain. This reduction of shear plane motion may be due to abnormal trunk movement patterns and/or intrinsic connective tissue pathology. There appears to be some sex-related differences in thoracolumbar fascia shear strain that may also play a role in altered connective tissue function.

## Background

The thoracolumbar fascia plays an important role in transferring forces among trunk muscles and the spine [1]. An important feature of this complex fascial structure is that it is composed of several layers of dense connective tissue separated by layers of "loose" areolar connective tissue that allow adjacent dense layers to glide past one another [2]. Independent motion of adjacent connective tissue layers is particularly relevant in structures such as the thoracolumbar fascia in which the dense layers correspond to the aponeuroses of muscles with different directions of pull: in this case, longitudinal (for latissimus dorsi, serratus posterior and erector spinae) vs. transverse (for internal/external obliques and latissimus dorsi).

Although the thoracolumbar fascia has been the subject of recent attention as a potential pain-generating structure in the back [3-6], its role in low back pain (LBP) pathophysiology is poorly understood. In a previous study using ultrasound, we found that human subjects with chronic LBP of more than 12 months duration had increased thickness and echogenicity of the perimuscular connective tissues forming the thoracolumbar fascia in the low back [6]. Abnormal connective tissue structure may be a predisposing factor for LBP, or a consequence of injury and/or changes in movement patterns occurring as a result of chronic pain. A potentially important consequence of injury may be fibrosis and adhesions, causing loss of independent motion of adjacent connective tissue layers which could further restrict body movements. Therefore, quantification of tissue mobility within the thoracolumbar fascia would be an important next step to investigate connective tissue pathophysiological alterations that may play a role in LBP.

Ultrasound elasticity imaging is a computational technique utilizing cross correlation methods to quantify tissue motion based on a series of ultrasound images acquired in rapid succession. In this study, we used a novel application of ultrasound elastography in which the relative mobility of layers within the thoracolumbar fascia was quantified in humans during passive trunk flexion induced by a motorized articulated table. Based on our previous findings of abnormal connective tissue structure in chronic LBP [6], we hypothesized that this relative motion would be reduced on average in a group of human subjects with chronic LBP of greater than 12 months duration compared with control subjects without low back pain (No-LBP). In addition, we compared thoracolumbar connective tissue motion to clinical tests commonly used during physical therapy to evaluate trunk range of motion and physical performance in LBP assessment.

## Methods

### Subjects and testing protocol

#### Human subject recruitment and selection criteria

The study was approved by the University of Vermont Institutional Review Board (CHRMS 07-025) and in compliance with the Helsinki Declaration. All subjects provided informed consent. Subjects were recruited by advertisements at the University of Vermont and associated facilities. The inclusion criterion for the LBP group was a history of recurrent or chronic LBP for at least 12 months as defined by Von Korff [7,8]. Recurrent LBP was defined as low back pain present on less than half the days in a 12-month period, occurring in multiple episodes over a year. Chronic LBP was defined as back pain present on at least half the days in a 12-month period. Inclusion criteria for No-LBP subjects were the absence of a history of low back pain or any other chronic pain that had limited activities of daily living or work and a numerical current pain index of less than 0.5 (on an 10 point Visual Analogue Scale). Additional exclusion criteria based on a subject's self report for both groups were: previous severe back or low extremity injury or surgery; major structural spinal deformity (scoliosis, kyphosis, stenosis) or spine surgery; ankylosing spondylitis or rheumatoid arthritis; spinal fracture, tumor or infection; clinical neurological deficit suggesting nerve root compression; neurological or major psychiatric disorder; bleeding disorders; corticosteroid medication or corticosteroid injection at L2-3 level of the back; pregnancy; worker's compensation or disability case; litigation for LBP; acute systemic infection. Subjects in the LBP group completed the McGill Pain questionnaire [9], the Oswestry Disability Scale questionnaire [10], as well as a custom-designed questionnaire about the onset, history and duration of their LBP. In addition, both groups completed the Baecke physical activity level questionnaire [11]. The Tampa Scale for Kinesiophobia was used to determine LBP subjects' level of fear toward movement in the presence of recurrent or chronic pain, with higher scores indicating heightened fear [12]. The Medical Outcomes Survey (MOS) was used as a general health, physical and mental quality of life measure for all subjects, with higher scores correlating with better health [13]. Subjects with No-LBP were frequency-matched to subjects with LBP for age, sex and body mass index (BMI) in order for the two groups to be balanced for these characteristics.

#### Testing protocol

**Figure 1:**
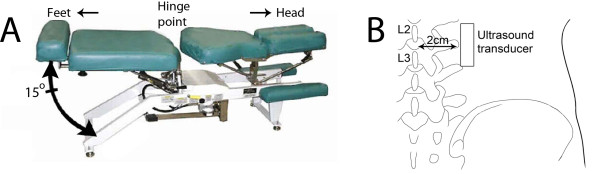
**Ultrasound image acquisition method**. A: Motorized articulated table capable of moving in the sagittal plane 15° at a rate of 0.5 Hz. The subject is positioned prone on the table with the hinge point at the L4-5 level. B: Location of ultrasound transducer (posterior view).

We tested 121 subjects, 71 with LBP and 50 with No-LBP. Each subject underwent a single testing session during which he/she was placed prone-lying on a motorized articulated table (Figure 1A). Use of a motorized table to passively move the trunk has the advantage of creating a reproducible rate and amplitude of input motion which is difficult to achieve with active trunk flexion. In addition, the prone position of the subject facilitated stabilization of the ultrasound probe on the skin. The subject was positioned such that the hinge point of the table was at the L4-5 interspace and the ultrasound transducer head was placed longitudinally 2 cm lateral to the midline at the level of the L2-3 interspace (Figure 1B). The rostral end of the transducer was fixed to the subject's skin using surgical tape, and the transducer was lightly stabilized by hand taking great care not to compress the tissues at any time during table motion. Lack of attachment at the caudal end allowed the skin to slide caudally during trunk flexion, while fixation at the rostral end prevented overall lateral and rostral translation of the ultrasound probe, which was verified during post processing. We used an ultrasound image field depth of 4 cm and a single ultrasound beam focal zone that was focused on the thoracolumbar fascia. This procedure was performed separately on the right and left sides of the back, with the order of testing randomized.

The motorized table underwent five cycles (0.5 Hz) of flexion with a range of 15° excursion for each cycle. During this table motion, we collected an ultrasound cine-loop (25 Hz) over a 10 second period using a Terason 3000 ultrasound machine equipped with a 10 MHz (12L5) linear array transducer. The ultrasound sampling rate was 25 MHz. The investigators performing the testing and ultrasound data analyses were blind to the subjects condition (LBP vs. No-LBP).

### Ultrasound measures

#### Ultrasound data post processing and thoracolumbar fascia tissue displacement calculation

**Figure 2:**
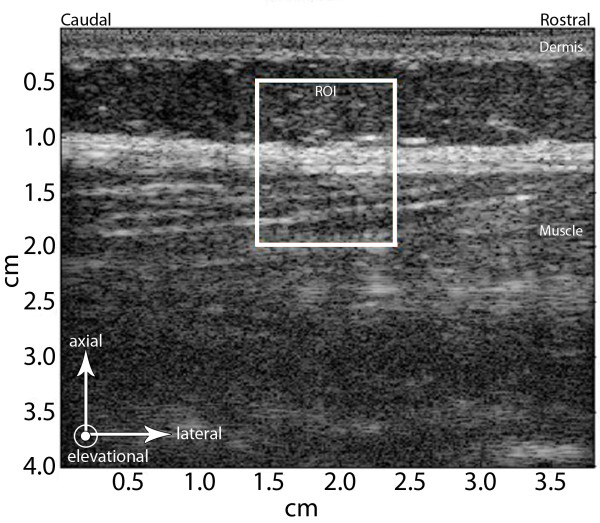
**Ultrasound elasticity imaging method**. White box indicates the region of interest (ROI) within the ultrasound image that was processed using cross correlation analyses. Arrows indicate reference axes within the ultrasound image: axial and lateral axes indicate directions parallel and perpendicular to the ultrasound beam respectively, in the plane of the ultrasound image. Elevational axis indicates direction perpendicular to the ultrasound image plane. Axial, lateral and elevational directions in the ultrasound image correspond to antero-posterior, rostro-caudal and medio-lateral anatomical directions respectively.

Ultrasound data from right and left sides were processed with a custom program written in Matlab (Natick, MA). Tissue displacements between successive ultrasound frames were estimated from the "raw" ultrasonic radio frequency (RF) data using cross-correlation techniques [14,15] with a 1 mm window incremented with a 90% overlap. The term "ultrasound frame" refers to the RF data acquired at each time point in the cine-loop. The terms "axial" and "lateral" indicate directions of tissue motion that are, respectively, along and perpendicular to the propagation of the ultrasound beam in the plane of the ultrasound image (Figure 2). The term "displacement" refers to the axial or lateral motion of the tissue between two successively acquired ultrasound frames (i.e. after 40 ms have elapsed). Tissue lateral displacement was computed for each successive pair of ultrasound frames in a 1 × 1.5 cm region of interest (ROI) centered laterally on the midpoint of the image and axially on the thoracolumbar fascia (Figure 2).

#### Thoracolumbar displacement and shear strain mapping

In order to visually document the presence of a shear plane within the thoracolumbar fascia, we generated successive displacement maps as a spatial representation of the displacement within the ROI for each pair of ultrasound frames. Corresponding cumulative lateral displacement maps were obtained by summing tissue displacements over time. Cumulative lateral shear strain maps were further generated by outputting the off-diagonal component in the Lagrangian finite strain tensor, which is obtained based on the displacement gradient [16].

#### Quantification of thoracolumbar shear strain at standardized location

**Figure 3:**
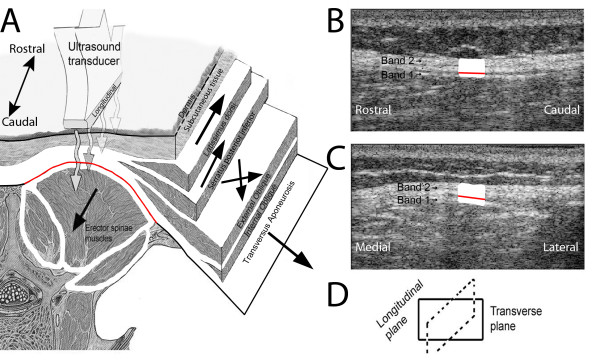
**Ultrasound imaging of thoracolumbar fascia**. A: Illustration of layers composing the thoracolumbar fascia corresponding to aponeuroses of back and abdominal wall muscles. Arrows indicate directions of pull for individual muscles. B-C: ultrasound image of thoracolumbar fascia in longitudinal (B) and transverse (C) planes showing echogenic (dense connective tissue) and echolucent (loose connective tissue) layers within the thoracolumbar fascia. A distinct echolucent plane (red line) is visible within the thoracolumbar fascia in the longitudinal image corresponding to the loose connective tissue layer located between the aponeurosis of the erector spinae muscles and the combined aponeuroses of the abdominal wall muscles, serratus posterior and latissimus dorsi.

To calculate the magnitude of shear deformation at a standardized location in human subjects with and without LBP, we used as a reference the echolucent plane separating the echogenic sheet closest to the erector spinae muscle (seen in longitudinal images as Band 1 in Figure 3B) from the more complex echogenic structure immediately superficial to it (Band 2 in Figure 3B).

**Figure 4:**
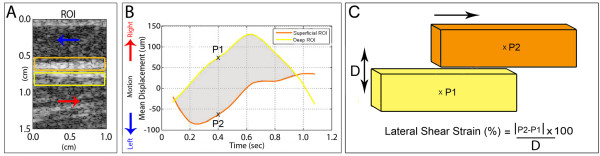
**Ultrasound data processing method**. A: Location of sub-ROIs (yellow and orange boxes) used for quantification of lateral tissue motion. B: Plot of lateral tissue displacement over time. Positive displacement in B corresponds to tissue movement toward the right (rostral, red arrows in A). Negative displacement in B corresponds to tissue movement toward the left (caudal, blue arrows in A). Yellow and orange lines in B respectively correspond to deep and superficial sub-ROIs in A. C: Shear strain model and calculation method. P1 and P2 represent the mean tissue displacement in the deep (yellow) and superficial (orange) Sub-ROIs respectively at each time point as shown in B. Shear strain between the sub-ROIs was calculated as the absolute difference in lateral motion between the superficial and deep sub-ROIs divided by the distance between the centers of the two sub-ROIs (2 mm) and expressed as a percentage.

With B-scan ultrasound, Band 1 is consistently visible as a thin echogenic line that moves with the underlying muscle and can thus be identified as the aponeurosis of the erector spinae muscle. In contrast, Band 2 is more variable in thickness, and sometimes contains one or more echogenic sub-bands which may correspond to the different aponeuroses that merge together to form the remainder of the thoracolumbar fascia (although this cannot be directly confirmed based solely on ultrasound). To calculate the magnitude of shear deformation at a standardized location in human subjects with and without LBP, we used as a reference the echolucent line separating Band 1 and Band 2 to define sub-regions of interest (sub-ROIs) each 2 mm × 10 mm (Figure 4A). The same blinded investigator identified the echogenic line in all images. Intra class correlation corresponding to intra-rater reliability for shear strain calculations (based on three separate measurements of six randomly selected images) was 0.98.

The cumulative lateral strain between superficial and deep sub-ROIs was calculated throughout one flexion cycle (shaded area in Figure 4B). P1 and P2 in Figure 4B represent the mean tissue displacement in the deep and superficial Sub-ROIs respectively at each time point. Shear strain between the sub-ROIs was calculated as the absolute difference in lateral motion between the superficial and deep sub-ROIs (|P2-P1| in Figure 4C) divided by the distance (2 mm) between the centers of the two sub-ROIs (D in Figure 4C) and expressed as a percentage. We used the absolute difference in lateral motion in order to quantify the total amount of shear strain deformation (both positive and negative) that occurs within the thoracolumbar fascia in response to passive trunk flexion. This shear strain calculation was repeated after shifting both sub-ROIs 0.5 mm superficially, then 0.5 mm deep to the original position. The maximum shear strain among the three positions was taken as the outcome measure for the right and left sides. The average of the two sides was used for statistical analysis.

#### Correction for axial tissue movement

Although the predominant thoracolumbar fascia tissue motion during passive trunk flexion is lateral (in the direction or red and blue arrows in Figure 4A), a small amount of axial tissue motion can also occur. In order to correct for any axial displacement, we used an automated tracking system that first determines the axial displacement map for each ultrasound frame pair. The location of the ROI at each time point was adjusted based on the mean axial displacement of the tissue relative to its starting position. The corrected sub-ROI positions were then used for determining lateral displacement.

#### Measurement of perimuscular connective tissue thickness and echogenicity

The thickness and echogenicity of the perimuscular connective tissues at the L2-3 level within the ROI was measured bilaterally by a blinded investigator as previously described [6]. Because the superficial border of the thoracolumbar fascia can merge with additional layers of subcutaneous connective tissue, this method uses operationally defined criteria based on the ultrasound intensity profile. First, perimuscular connective tissue thickness was defined as the thickness of the echogenic layered structure located closest to the muscle and separated from the nearest, more superficial echogenic layer by more than 2 mm. Second, perimuscular connective tissue echogenicity was defined as the area under the curve of the ultrasound intensity profile within the portion of the ROI delineated by the perimuscular thickness measurements. Ultrasound measurements were made on images reconstituted from raw ultrasonic data in Matlab software (The MathWorks, Natick, MA) using a Hilbert transformation without additional image enhancements.

### Clinical measures

#### Range of motion and physical performance measures

A number of clinical tests commonly used during physical therapy LBP assessments were performed to evaluate trunk range of motion and physical performance. These measures may be affected by both tissue abnormalities (e.g. increased stiffness) and pain; therefore, these measures were used in this study to 1) begin to understand the impact of connective tissue abnormalities on overall function and 2) plan future studies that combine functional assessment with more specific measurements of tissue behavior during active and passive trunk motion.

In the physical performance measures, subjects performed active trunk movements and tasks; the time necessary to perform these tasks was recorded in seconds using a stop watch. Given that these tests were secondary outcome measures, the number of tests was kept to a minimum in order to avoid excessive fatigue or soreness prior to ultrasound testing; the subject was also instructed not to move into ranges of motion that caused increased discomfort in the low back region. Range of motion tests were performed first, followed by performance tests.

#### Trunk range of motion (ROM) measurements

We used the double inclinometer technique for measurement of lumbar flexion [17], extension and lateral flexion [18] ROM. While the subject stood erect, an inclinometer (a circular, fluid-filled instrument with a weighted needle that indicates the number of degrees on a protractor scale) was placed on the dorsal midline at the level of L1-2 interspinous space (upper) and at the level of the posterior superior iliac crests (PSIS) (lower). The inclinometers were "zeroed" and the subject was instructed to flex his/her trunk forward as far as he/she could without bending the knees. The examiner recorded the number of degrees on both the upper and lower inclinometers. The amount of motion of the lower inclinometer was subtracted from the upper inclinometer to derive the total lumbar spine flexion (lumbar flexion ROM). A similar procedure was used to record the lumbar extension ROM. For males and females respectively, the normal ROM is 65.0 and 64.4 degrees for trunk flexion and the normal ROM is 26.6 and 27.3 degrees for trunk extension [19]. For lateral flexion ROM (performed bilaterally), the inclinometers were placed in the same locations and oriented in the frontal plane (rather than the sagittal plane for measuring flexion/extension). The same subtraction of the upper minus lower inclinometer readings provided the total lumbar lateral flexion ROM. The normal ROM for lateral flexion is 24 degrees [18].

#### Functional measures (task duration)

##### Repeated Trunk Flexion test

From a neutral standing position, the subject maximally flexed his/her trunk forward and returned to the upright position as fast as comfortably tolerated. The total time (sec) to complete five repetitions of trunk flexion/extension was recorded.

##### Repeated sit-to-stand test

From a standardized seated position, the subject rose to standing and returned to sitting as quickly as possible five times. The total time (sec) taken to complete five repetitions was recorded.

##### 50-ft walk test

Subjects walked 50 feet, first as fast as they could and then at their preferred walking speed. The total time (sec) to complete the fast and self selected walk was recorded.

##### Sorrensen's test

To assess trunk muscle strength and endurance, subjects were positioned prone on a table such that only their lower limbs and pelvis were supported on the table top. While their lower body was stabilized by the examiner, the subject was asked to contract his/her trunk extension muscles to maintain a horizontal trunk position against gravity while unsupported. The total time (sec) holding the trunk horizontal without dropping below 10 degrees to the horizontal was recorded.

### Statistical methods

A chi square test was used to compare LBP and No-LBP groups on the distribution of males and females. Two-way analyses of variance and covariance were used to compare LBP and No-LBP groups on continuous outcomes with sex as the additional factor in the model. For outcomes in which BMI was a significant predictor, significance levels were based on analyses of covariance with corresponding means representing least square means adjusted for the covariate. If there was evidence that group comparisons were different across males and female (i.e. group by sex interaction, p-value < .10), group comparisons within sex were based on Fisher's Least Square Difference (LSD) procedure. The associations between shear strain and other outcomes were evaluated using Spearman's rank correlations. All statistical analyses were performed using SAS Statistical Software Version 9.2 (SAS Institute, Cary, NC). For those outcomes measured bilaterally (thoracolumbar fascia shear strain, perimuscular connective tissue thickness, perimuscular connective tissue echogenicity, lateral trunk flexion ROM), analyses reported represent the average of the right and left sides. Results of analyses performed within right and left sides paralleled the overall findings. The type 1 error rate was set at α = .05 on a comparison wise basis.

## Results

The percentage of male subjects in the LBP and No-LBP groups was 53% and 48% respectively (chi square = 0.36, p = .55). There were no significant differences between LBP and No-LBP groups for age, (44.6 ± 1.8 vs. 41.8 ± 2.3, p = .35), BMI (26.0 ± 0.5 vs. 26.1 ± 0.6, p = .76), and activity levels measured by the Baecke Activity index (8.0 ± 0.3 vs. 7.7 ± 0.5, p = .61). There also were no significant differences between groups for age, BMI and activity level within either males or females. Indices of symptom severity and disability in subjects with LBP are shown in Table 1.

**Table 1:** Indices of symptom severity and disability in subjects with LBP

		Males	Females	p-value
McGill pain questionnaire (# of words circled)		7.1 ± 0.5	8.2 ± 0.9	p = .31
Duration of pain (years)		12.9 ± 1.7	13.5 ± 2.5	p = .83
Pain level (0-10 Scale)		2.8 ± 0.4	3.5 ± 0.4	p = .24
Current pain intensity on day of testing (0-10 scale)		1.5 ± 0.3	2.5 ± 0.4	p = .053
Exacerbation intensity (0-10 scale)		6.1 ± 0.4	5.2 ± 0.4	p = .17
Exacerbation frequency (%)	Yearly	23	3	p = .01
	Monthly	20	32	
	Weekly	14	39	
	Daily	43	26	
Exacerbation duration (days)		50.1 ± 21.5	39. 9 ± 20.9	p = .73
Initial injury (%)		33	48	p = .20
Oswestry	Mild (0-20)	71	58	p = .56
disability	Moderate (21-40)	26	39	
scale (%)	Severe (>40)	3	3	
TAMPA kinesiophobia scale		39.9 ± 0.9	35.1 ± 1.0	p < .001
Von Korff (%)	Recurrent	42	45	p = .77
	Chronic	58	55	

Note. All measures were reported via take-home questionnaires except the current pain intensity measure which was reported on the day of testing. Values represent Mean ± SE unless otherwise indicated.

The following two video clips show examples of thoracolumbar fascia motion during passive trunk flexion in a human subject with No-LBP (Additional file 1) and a subject with LBP (Additional file 2). In the subject with No-LBP, the layers within the thoracolumbar fascia can be seen to move independently with some adjacent layers moving in opposite directions. In contrast, in the subject with LBP, there is less apparent differential motion between the adjacent layers.

**Figure 5:**
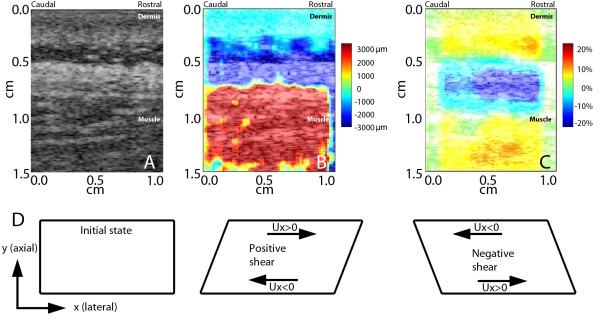
**Cumulative lateral tissue displacement and shear strain maps**. A: B-scan ultrasound image ROI. B: Sum of tissue displacement over time (cumulative displacement) during one flexion cycle of the table within the ultrasound image ROI. Red indicates tissue displacement toward the right (rostral) and blue indicates tissue displacement toward the left (caudal). C: Cumulative shear strain within the ultrasound image ROI. Red and blue indicate positive (toward the right) and negative (toward the left) shear strain respectively. (B) and (C) respectively correspond to cumulative tissue displacement and shear strain at the end of one flexion cycle of the motorized table. D: Diagram illustrating positive and negative shear strains which represent sliding or deformation of an object in different directions. The shear component is obtained by taking the gradient of lateral displacement (Ux) along the positive axial direction (+y). The x-y coordinates are defined corresponding to the ultrasound imaging configuration (see axes in Figure 2).

The next two video clips (Additional file 3 and Additional file 4) respectively show cumulative lateral displacement and corresponding shear strain maps within the ROI during one flexion cycle of the table. In both movies, red indicates tissue displacement or shear strain toward the right (rostral) and blue indicates tissue displacement or shear strain toward the left (caudal). Figures 5A, B and 5C respectively show B-scan, cumulative displacement and cumulative shear strain maps at the end of one flexion cycle of the motorized table demonstrating the presence of shear plane deformation within the thoracolumbar fascia as illustrated in Figure 5D.

**Figure 6:**
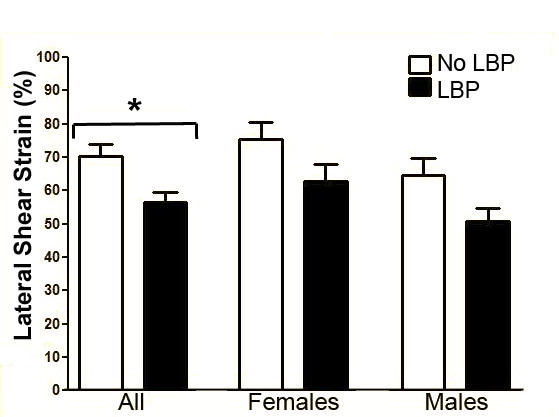
**Thoracolumbar shear strain in human subjects with and without LBP**. Thoracolumbar shear strain was ~20% lower in human subjects with chronic LBP compared with No-LBP. *indicates p < .01. N = 121 subjects. Error bars represent standard errors.

When shear strain was calculated using anatomically defined locations as shown in Figure 4, average shear strain was 62% (SD = 27.2%) among all subjects tested. On average, thoracolumbar fascia shear strain was 20% lower in subjects with LBP compared with subjects without LBP. For the LBP vs. No-LBP groups, thoracolumbar fascia shear strain was (mean±SE) 56.4% ± 3.1% vs. 70.2% ± 3.6% respectively, p < .01) (Figure 6). There was no evidence that this difference was sex-specific (group by sex interaction p = .09) although overall, males had significantly lower shear strain than females (p = .02). There were no significant overall correlations between thoracolumbar fascia shear strain and either age (r = -0.18, p = .06), BMI (r = -0.13, p = .16) or activity level (r = -0.09, p = .34). Additionally, in subjects with LBP, there were no significant correlations between thoracolumbar fascia shear strain and responses to McGill pain questionnaire (r = 0.03, p = .84), pain level (r = 0.03, p = .81), pain intensity on day of testing (r = 0.01, p = .93) or Oswestry disability scale (r = 0.12. p = .34). However, thoracolumbar fascia shear strain was negatively correlated with pain duration in males with LBP (r = -0.46, p < .0004) but not in females (r = -0.07, p = .67).

Results of testing for perimuscular connective tissue thickness and echogenicity, trunk range of motion and functional measures for male and female subjects are shown in Table 2. Significant differences were found between the two groups for several outcome measures: flexion range of motion, extension range of motion and Sorrensen's endurance test were decreased in the LBP group while perimuscular connective tissue echogenicity, repeated trunk flexion task duration, repeated sit to stand task duration and 50 foot walk task duration (regular and fast pace) were increased in the LBP group. Some of the outcome measures (perimuscular connective tissue thickness, extension range of motion, repeated sit-to-stand task duration and Sorrensen's endurance test) were gender-specific (see letter superscripts in Table 2).

**Table 2:** Outcome Measures for Male and Female Subjects with and without Low Back Pain

	Males		Females				
Outcomes	No-LBP (n = 24)	LBP (n = 38)	No-LBP (n = 26)	LBP (n = 33)	Group p-value	Sex p-value	Group by Sex p-value
Percent Shear Strain	64.70 ± 5.17	50.88 ± 3.77	75.36 ± 5.02	62.73 ± 5.18	.007	.02	.90
Perimuscular Thickness*	0.37 ± 0.04^a^	0.49 ± 0.03^b^	0.41 ± 0.03^a^	0.41 ± 0.03^a^	.07	.50	.09
Perimuscular Echogenicity*	0.13 ± 0.01	0.16 ± 0.01	0.14 ± 0.01	0.15 ± .01	.007	.92	.30
Flexion Range of Motion	53.90 ± 1.88	46.38 ± 2.27	53.58 ± 1.84	52.13 ± 1.78	.03	.19	.15
Extension Range of Motion	16.90 ± 1.89^a^	9.74 ± 0.86^b^	16.89 ± 1.55^a^	16.28 ± 1.92^a^	.02	.04	.04
Lateral Flexion	19.51 ± 0.68^a^	17.07 ± 0.61^b^	18.02 ± 0.67^a^	18.23 ± 0.61^a^	.09	.80	.04
Repeated Trunk Flexion*	7.97 ± 0.52	9.88 ± 0.41	8.27 ± 0.45	9.64 ± 0.41	<.001	.94	.56
Repeated Sit to Stand	10.71 ± 0.43^a^	13.42 ± 0.62^b^	11.90 ± 0.54^a^	12.67 ± 0.41^a^	.002	.69	.08
50 Foot Walk Regular Speed*	10.64 ± 0.35	11.58 ± 0.27	11.19 ± 0.30	11.97 ± 0.28	.005	.12	.80
50 Foot Walk Fast Speed*	6.82 ± 0.26	7.56 ± 0.20	7.25 ± 0.22	8.24 ± 0.21	<.001	.71	.57
Sorensen's*	126.5 ± 10.1^a^	104.9 ± 7.9^a^	139.2 ± 8.9^a^	85.7 ± 8.1^b^	<.001	.71	.08

Note: tabled values are mean ± SE unless otherwise indicated. Variables denoted by an asterisk indicate values are least square mean ± SE, which are adjusted for BMI. For those variables in which there was evidence that differences between LBP and No LBP were dependent on sex (i.e. group by sex interaction p <.10), group comparisons were performed within males and females. Superscripts a and b indicate that group means not sharing a common letter are significantly different within each sex (Fisher's LSD, p <.05).

Significant correlations were found in male subjects between thoracolumbar fascia shear strain and perimuscular connective tissue thickness (r = -0.45, p < .001), echogenicity (r = -0.28, p < .05), trunk flexion range of motion (r = 0.36, p < .01), trunk extension range of motion (r = 0.41, p < .01), repeated forward bend task duration (r = -0.54, p < .0001) and repeated sit-to-stand task duration (r = -0.45, p < .001). No significant correlations were found in females between thoracolumbar fascia shear strain and any of these outcome measures. There were also no significant correlations in either males or females between thoracolumbar fascia shear strain and measures of anxiety, cognitive function, mental health or psychological distress (MOS questionnaire) or kinesiophobia (Tampa questionnaire).

## Discussion

This study reports the first quantitative evaluation of shear strain within the thoracolumbar fascia in humans. Mapping of shear strain using elastography, as well as computation of shear strain using anatomically defined sub-ROIs demonstrated the presence of a prominent shear plane at the first echolucent plane superficial to the muscle/fascia interface. We found that, during a standardized passive flexion test, shear strain was reduced by ~20% in a group of human subjects with chronic LBP.

The lack of correlation between thoracolumbar fascia shear strain and subjective psychosocial outcome measures (including pain level) suggests that reduced shear strain may not correlate with pain symptoms over time. However, thoracolumbar fascia shear strain may nevertheless be a useful biomarker for pathophysiological processes that may predispose to chronic LBP or may influence its long term trajectory including the increased likelihood of recurrence, especially in males in whom we found a moderate positive correlation between shear strain and LBP duration. This, and the additional male-specific moderate correlations with connective tissue thickness and echogenicity, range of motion and physical function, could be related to body composition, fat distribution pattern, hormonal factors, or to structural and/or movement pattern differences between males and females. The latter explanation is supported by previous reports of specific lumbopelvic movement impairment in males with low back pain [20,21]. In the current study, we found that differences in perimuscular connective tissue thickness between LBP and No-LBP were only significant in males. In our prior report [22], we did not have evidence that this difference was sex-specific, although we had observed a greater difference between LBP and No-LBP in males. We did however confirm our previous finding that perimuscular connective tissue echogenicity is greater in LBP in both males and females. If differences in connective tissue thickness are indeed limited to males, this could be related to some of the other male-specific findings observed in this study such as decreased range of motion and functional measures.

A limitation of this study is that measurements of thoracolumbar fascia shear strain were made only at the L2-3 level. This was chosen in this initial study because, at this location, the skin surface is relatively flat and the thoracolumbar fascia is relatively parallel to the skin, which simplifies calculation of lateral displacement. Applying this technique to more caudal low back segments, as well as other body regions where restricted mobility between adjacent connective tissue planes may be present, could potentially contribute to a more general understanding of the role of connective tissue in chronic pain pathophysiology [23]. This measurement method could also be adapted to active, as opposed to passive, body movements although this would pose additional challenges for stabilization of the ultrasound probe.

Given that the dense connective tissue layers within the thoracolumbar fascia are aponeuroses connected to dorsal and ventral trunk muscles, one plausible explanation for our findings is that reduced shear strain results from impaired neuromuscular control and recruitment patterns of these muscles during trunk movements which has been shown to be associated with chronic LBP [24-26]. Alternatively, the altered muscle recruitment patterns could lead to altered forces being transferred to the connective tissues, which could cause remodeling as can occur in other types of connective tissues such as ligaments and joint capsules [27-33]. Over time, the altered movement patterns could worsen connective tissue adhesions resulting in increased movement restriction, especially in the presence of pain and inflammation. A third possibility is that reduction of shear strain could be due to intrinsic connective tissue pathology (e.g. chronic inflammation, fibrosis) resulting from direct injury to the connective tissue. Concurrent measurement of shear strain and electromyographic measurement of muscle activity will be an important next step to further understand these potentially important pathophysiological mechanisms. Such studies may lead to defining a subgroup of patients with decreased shear plane motion predominantly due to abnormal movement strategy who may benefit from movement reeducation, versus a subgroup with decreased shear plane mobility due to fibrosed connective tissue layers who may benefit from direct connective tissue manipulation.

## Conclusions

In summary, thoracolumbar fascia shear strain was reduced in a group of human subjects with LBP of greater than 12 months duration compared to a control group with No-LBP. Although differences in thoracolumbar fascia shear strain between LBP and No-LBP were found in both sexes, shear strain was lower in males overall, and significant correlations with trunk flexibility, functional measures and connective tissue structure were found in males only. Possible explanations for reduced thoracolumbar fascia shear strain during passive trunk flexion in LBP include abnormal patterns of trunk muscle activity and/or intrinsic connective tissue pathology.

## Abbreviations

(LBP): Low back pain; (No-LBP): No low back pain; (ROM): Range of motion; (ROI): Region of interest.

## Competing interests

The authors declare that they have no competing interests.

## Authors' contributions

HML conceived the study, participated in study design and human subject testing and drafted the manuscript; JRF and WNL developed and implemented the ultrasound analysis methods; GJB performed statistical analyses; CK recruited the subjects; CK and NAB participated in testing of subjects and manuscript preparation; AGN performed physical therapy assessments and SMH, EEK and JJT participated in study design. All authors read, edited and approved the final manuscript.

## Pre-publication history

The pre-publication history for this paper can be accessed here:

http://www.biomedcentral.com/1471-2474/12/203/prepub

## Supplementary Material

Additional file 1 **Video clip of thoracolumbar fascia motion in human subject with No-LBP**. Ultrasound B-scan acquired during passive trunk flexion induced by a motorized articulated table. Ultrasound transducer is placed longitudinally 2 cm from the midline at the level of the L2-3 interspace. Click here for file

Additional file 2 **Video clip of thoracolumbar fascia motion in human subject with LBP**. Ultrasound B-scan acquired during passive trunk flexion induced by a motorized articulated table. Ultrasound transducer is placed longitudinally 2 cm from the midline at the level of the L2-3 interspace. Click here for file

Additional file 3 **Video clip of cumulative lateral displacement map during one flexion cycle of the table**. Red indicates tissue displacement toward the right (rostral) and blue indicates tissue displacement or shear strain toward the left (caudal) (see color scales in Figure 5). Click here for file

Additional file 4 **Video clip of cumulative lateral shear strain map during one flexion cycle of the table**. Red indicates shear strain toward the right (rostral) and blue indicates shear strain toward the left (caudal) (see color scales in Figure 5). Click here for file
